# 1-(2-Hy­droxy­eth­yl)-4-{3-[(*E*)-2-(trifluoro­meth­yl)-9*H*-thioxanthen-9-yl­idene]prop­yl}piperazine-1,4-diium bis­(3-carb­oxy­prop-2-enoate)

**DOI:** 10.1107/S160053681102722X

**Published:** 2011-07-13

**Authors:** M. S. Siddegowda, Ray J. Butcher, Mehmet Akkurt, H. S. Yathirajan, A. R. Ramesh

**Affiliations:** a Department of Studies in Chemistry, University of Mysore, Manasagangotri, Mysore 570 006, India; b Department of Chemistry, Howard University, 525 College Street NW, Washington, DC 20059, USA; c Department of Physics, Faculty of Sciences, Erciyes University, 38039 Kayseri, Turkey; d R. L. Fine Chem, Bangalore 560 064, India

## Abstract

In the title salt, C_23_H_27_F_3_N_2_OS^+^·2C_4_H_3_O_4_^−^, a non-merohedral twin [ratio of the twin components = 0.402 (1):0.598 (1)], the –CF_3_ group is disordered over two sets of sites with occupancy factors in the ratio 0.873 (2):0.127 (2). The dihedral angle between the two outer aromatic rings of the 9*H*-thioxanthene unit, whose thio­pyran ring has a screw-boat conformation, is 33.01 (9)°. The diprotonated piperazine ring adopts a chair conformation. In the crystal, inter­molecular O—H⋯O, N—H⋯O and C—H⋯O hydrogen bonds between neighboring mol­ecules form zigzag chains along the *a* axis and contribute to the stabilization of the packing.

## Related literature

The title compound was formed by the reaction of flupentixol (systematic name: 2-[4-[3-[(*EZ*)-2-(trifluoro­meth­yl)-9*H*-thio­xanthen-9-yl­idene]prop­yl]piperazin-1-yl]ethanol and fumaric acid. For the anti­depressant action of flupentixol, see: Robertson & Trimble, (1981 ▶). For related structures, see: Post *et al.* (1975*a* ▶,*b* ▶); Jones *et al.* (1977 ▶). For ring puckering parameters, see: Cremer & Pople (1975 ▶).
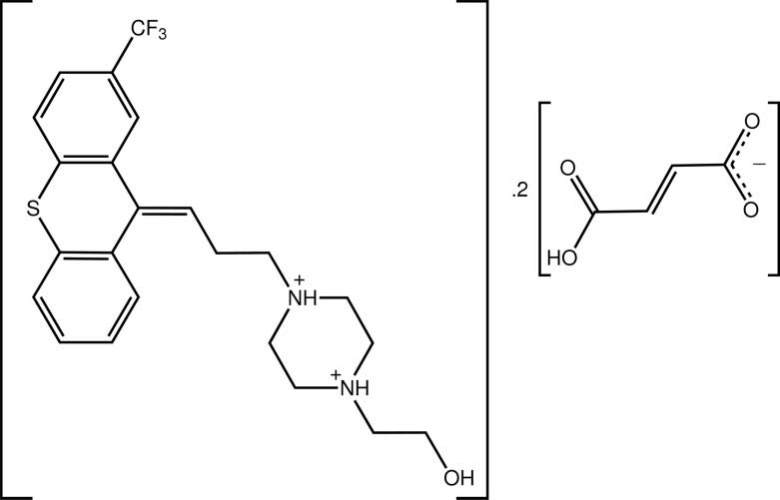


## Experimental

### 

#### Crystal data

C_23_H_27_F_3_N_2_OS^2+^·2C_4_H_3_O_4_^−^
*M* *_r_* = 666.66
Triclinic, 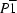

*a* = 6.4175 (2) Å
*b* = 9.6185 (4) Å
*c* = 25.5771 (10) Å
α = 96.377 (4)°
β = 96.295 (3)°
γ = 92.774 (3)°
*V* = 1556.63 (10) Å^3^
*Z* = 2
Cu *K*α radiation
μ = 1.59 mm^−1^
*T* = 295 K
0.53 × 0.17 × 0.12 mm

#### Data collection

Oxford Diffraction Xcalibur Ruby Gemini diffractometer
Absorption correction: multi-scan (*CrysAlis PRO*; Oxford Diffraction, 2007 ▶) *T*_min_ = 0.643, *T*_max_ = 1.000
11625 measured reflections
11625 independent reflections
9926 reflections with *I* > 2σ(*I*)

#### Refinement

*R*[*F* ^2^ > 2σ(*F* ^2^)] = 0.055
*wR*(*F* ^2^) = 0.162
*S* = 1.03
11625 reflections
430 parameters
12 restraints
H-atom parameters constrained
Δρ_max_ = 0.47 e Å^−3^
Δρ_min_ = −0.27 e Å^−3^

### 

Data collection: *CrysAlis PRO* (Oxford Diffraction, 2007 ▶); cell refinement: *CrysAlis PRO*; data reduction: *CrysAlis RED* (Oxford Diffraction, 2007 ▶); program(s) used to solve structure: *SHELXS97* (Sheldrick, 2008 ▶); program(s) used to refine structure: *SHELXL97* (Sheldrick, 2008 ▶); molecular graphics: *ORTEP-3 for Windows* (Farrugia, 1997 ▶); software used to prepare material for publication: *WinGX* (Farrugia, 1999 ▶) and *PLATON* (Spek, 2009 ▶).

## Supplementary Material

Crystal structure: contains datablock(s) global, I. DOI: 10.1107/S160053681102722X/tk2763sup1.cif

Structure factors: contains datablock(s) I. DOI: 10.1107/S160053681102722X/tk2763Isup2.hkl

Supplementary material file. DOI: 10.1107/S160053681102722X/tk2763Isup3.cml

Additional supplementary materials:  crystallographic information; 3D view; checkCIF report

## Figures and Tables

**Table 1:** Hydrogen-bond geometry (Å, °)

*D*—H⋯*A*	*D*—H	H⋯*A*	*D*⋯*A*	*D*—H⋯*A*
O1—H1⋯O1*B*^i^	0.82	2.05	2.8365 (16)	162
N1—H1*A*⋯O1*A*	0.91	1.81	2.7055 (15)	168
N1—H1*A*⋯O2*A*	0.91	2.57	3.2580 (15)	133
N2—H2*A*⋯O1*B*^i^	0.91	1.86	2.7572 (15)	167
N2—H2*A*⋯O2*B*^i^	0.91	2.52	3.2230 (15)	134
O4*A*—H4*A*⋯O2*A*^ii^	0.82	1.73	2.5406 (16)	167
O4*B*—H4*B*⋯O2*B*^i^	0.82	1.74	2.5497 (16)	168
C2*A*—H2*AA*⋯O3*A*	0.93	2.51	2.8251 (17)	100
C2*B*—H2*BA*⋯O3*B*	0.93	2.50	2.8165 (17)	100
C16—H16*B*⋯O3*A*^iii^	0.97	2.56	3.2782 (19)	131
C17—H17*B*⋯O4*A*^iv^	0.97	2.59	3.4520 (19)	148
C19—H19*A*⋯O2*A*	0.97	2.57	3.2871 (18)	131
C19—H19*B*⋯O1*A*^i^	0.97	2.41	3.2461 (17)	144
C20—H20*A*⋯O1^v^	0.97	2.44	3.3877 (18)	167
C21—H21*A*⋯O1*B*	0.97	2.41	3.2098 (16)	140
C21—H21*B*⋯O2*B*^i^	0.97	2.51	3.2367 (17)	132
C22—H22*B*⋯O3*B*^vi^	0.97	2.51	3.3848 (18)	150
C22—H22*B*⋯O4*B*^vi^	0.97	2.55	3.4236 (18)	150

Symmetry codes: (i) 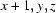; (ii) 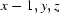; (iii) 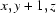; (iv) 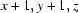; (v) 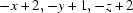; (vi) 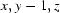.

**Fig. 1.**
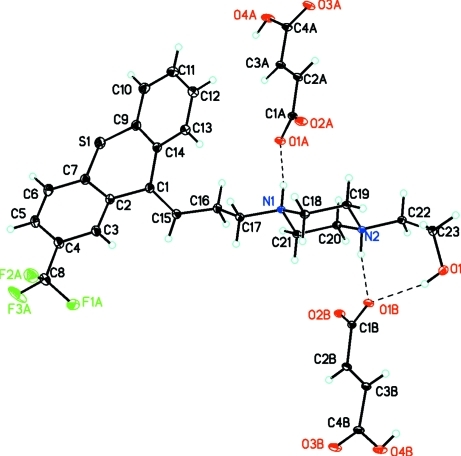
Molecular structure of (I) with the atom labeling scheme. Displacement ellipsoids for non-H atoms are drawn at the 30% probability level. Hydrogen bond connections between the components of the asymmetric unit are shown as dashed lines. Only the major orientation of the disordered CF3 group is shown.

**Fig. 2.**
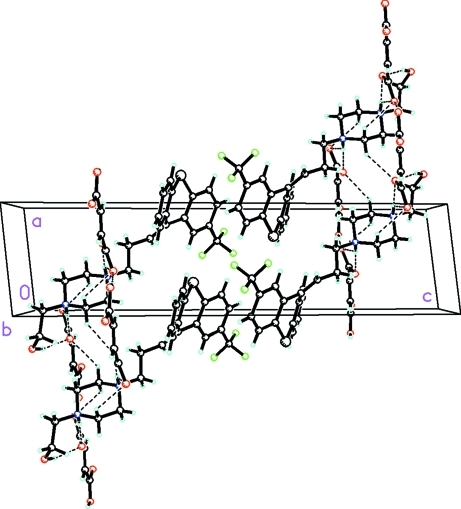
Perspective view of the crystal packing and hydrogen bonding (dashed lines) of (I) down the b axis.

## supplementary crystallographic information

### Comment

Flupentixol (systematic IUPAC name: 2-[4-[3-[(*EZ*)-2-(trifluoromethyl)-9*H*-thioxanthen-9-ylidene] propyl]piperazin-1-yl]ethanol is a typical antipsychotic drug of the thioxanthene class. In addition to pure drug preparations, it is also available as deanxit, a combination product containing both melitracen and flupentixol.The antidepressant action of flupentixol has been described (Robertson & Trimble, 1981). The crystal structures of α-flupenthixol (Post *et al.*, 1975*b*), β-flupenthixol (Post *et al.*, 1975*a*) and piflutixol (Jones *et al.*, 1977) have been reported. In view of the importance of flupentixol, this paper reports the crystal structure of the title salt, (I), C_23_H_27_F_3_N_2_^+^OS_2_.2[C_4_H_3_O_4_^-^], formed by the reaction of flupentixol and fumaric acid.

The crystal studied was a non-merohedral twin, the ratio of the twin components being 0.402 (1): 0.598 (1). In (I), Fig. 1, the thiopyran ring of the 9*H*-thioxanthene ring system (S1/C1/C2/C7C9/C14) has a screw-boat conformation: the puckering parameters (Cremer & Pople, 1975) of Q_T_ = 0.5118 (15) Å, θ = 87.53 (19)° and φ = 3.2 (2) °. The dihedral angle between the two outer aromatic rings of the 9*H*-thioxanthene unit is 33.01 (9) °. The diprotonated piperazine ring (N1/N2/C18–C21) adopts a chair conformation with puckering parameters Q_T_ = 0.580 (13) Å, θ = 178.76 (12) ° and φ = 177 (7) °.

In the crystal structure, intermolecular O—H···O, N—H···O and C—H···O hydrogen bonds (Table 1) contributes to crystal packing forming the zigzag chains parallel to the [100] direction (Fig. 2).

### Experimental

Flupenthixol base (2.0 g, 0.046 mol) was dissolved in 10 ml of ethyl acetate and fumaric acid (1.067 g, 0.092 mol) was added at 323 K. The solution was stirred in a round bottomed flask at 343 K for 30 min. The mixture was cooled to room temperature and the product formed was filtered and dried. X-ray quality crystals were obtained from a 1:1 mixture of dichloromethane and methanol by slow evaporation (*M*.pt.: 431-433 K).

### Refinement

All H atoms were positioned geometrically and allowed to ride on their parent atoms, with C—H = 0.93–0.97 Å, N–H = 0.91 Å and O–H = 0.82 Å, and with *U*_iso_(H) = 1.5*U*_eq_(O) for hydroxyl H atoms and 1.2*U*_eq_(C) for other H atoms. The CF_3_ group is disordered over two sets of sites with occupations of 0.873 (2) and 0.127 (2), respectively. The structure was refined as a non-merohedral twin (using HKLF 5 in SHELXL) with the final ratio of the twin components being 0.402 (1):0.598 (1); as such there is no value for *R*_int_.

### Crystal data

**Table:**

C_23_H_27_F_3_N_2_OS^2+^·2C_4_H_3_O_4_^−^	*Z* = 2
*M**_r_* = 666.66	*F*(000) = 696
Triclinic, *P*1	*D*_x_ = 1.422 Mg m^−^^3^
Hall symbol: -P 1	Cu *K*α radiation, λ = 1.54184 Å
*a* = 6.4175 (2) Å	Cell parameters from 5369 reflections
*b* = 9.6185 (4) Å	θ = 4.6–75.4°
*c* = 25.5771 (10) Å	µ = 1.59 mm^−^^1^
α = 96.377 (4)°	*T* = 295 K
β = 96.295 (3)°	Thick needle, colourless
γ = 92.774 (3)°	0.53 × 0.17 × 0.12 mm
*V* = 1556.63 (10) Å^3^	

### Data collection

**Table:**

Oxford Diffraction Xcalibur Ruby Gemini diffractometer	11625 independent reflections
Radiation source: Enhance (Cu) X-ray Source	9926 reflections with i > 2σ(i)
graphite	*R*_int_ = 0.000
Detector resolution: 10.5081 pixels mm^-1^	θ_max_ = 75.7°, θ_min_ = 4.6°
ω scans	*h* = −8→5
Absorption correction: multi-scan (*CrysAlis PRO*; Oxford Diffraction, 2007)	*k* = −11→12
*T*_min_ = 0.643, *T*_max_ = 1.000	*l* = −31→31
11625 measured reflections	

### Refinement

**Table:**

Refinement on *F*^2^	Primary atom site location: structure-invariant direct methods
Least-squares matrix: full	Secondary atom site location: difference Fourier map
*R*[*F*^2^ > 2σ(*F*^2^)] = 0.055	Hydrogen site location: inferred from neighbouring sites
*wR*(*F*^2^) = 0.162	H-atom parameters constrained
*S* = 1.03	*w* = 1/[σ^2^(*F*_o_^2^) + (0.0915*P*)^2^ + 0.2879*P*] where *P* = (*F*_o_^2^ + 2*F*_c_^2^)/3
11625 reflections	(Δ/σ)_max_ = 0.001
430 parameters	Δρ_max_ = 0.47 e Å^−^^3^
12 restraints	Δρ_min_ = −0.27 e Å^−^^3^

### Special details

**Table:**

Geometry. Bond distances, angles *etc*. have been calculated using the rounded fractional coordinates. All su's are estimated from the variances of the (full) variance-covariance matrix. The cell e.s.d.'s are taken into account in the estimation of distances, angles and torsion angles
Refinement. Refinement on *F*^2^ for ALL reflections except those flagged by the user for potential systematic errors. Weighted *R*-factors *wR* and all goodnesses of fit *S* are based on *F*^2^, conventional *R*-factors *R* are based on *F*, with *F* set to zero for negative *F*^2^. The observed criterion of *F*^2^ > σ(*F*^2^) is used only for calculating -*R*-factor-obs *etc*. and is not relevant to the choice of reflections for refinement. *R*-factors based on *F*^2^ are statistically about twice as large as those based on *F*, and *R*-factors based on ALL data will be even larger.

### Fractional atomic coordinates and isotropic or equivalent isotropic displacement parameters (Å^2^)

**Table:**

	*x*	*y*	*z*	*U*_iso_*/*U*_eq_	Occ. (<1)
S1	−0.28261 (7)	0.43649 (6)	0.60072 (2)	0.0659 (2)	
F1A	0.4612 (4)	0.9150 (2)	0.57634 (7)	0.1255 (9)	0.873 (2)
F2A	0.4440 (3)	0.8145 (2)	0.49990 (8)	0.0980 (7)	0.873 (2)
F3A	0.2283 (3)	0.9651 (2)	0.51652 (13)	0.1469 (15)	0.873 (2)
O1	1.32172 (19)	0.39302 (13)	0.98491 (5)	0.0546 (4)	
N1	0.66238 (16)	0.39316 (11)	0.79768 (4)	0.0305 (3)	
N2	0.91809 (17)	0.37193 (11)	0.89824 (4)	0.0306 (3)	
C1	0.1779 (2)	0.48863 (17)	0.64991 (6)	0.0438 (4)	
C2	0.1109 (3)	0.56962 (18)	0.60557 (6)	0.0477 (5)	
C3	0.2459 (3)	0.66960 (18)	0.58916 (7)	0.0516 (5)	
C4	0.1775 (3)	0.7513 (2)	0.55001 (8)	0.0582 (6)	
C5	−0.0262 (3)	0.7307 (2)	0.52472 (8)	0.0656 (7)	
C6	−0.1606 (3)	0.6297 (2)	0.53930 (8)	0.0651 (7)	
C7	−0.0948 (3)	0.55110 (19)	0.58004 (7)	0.0521 (5)	
C8	0.3238 (3)	0.8631 (2)	0.53587 (8)	0.0680 (7)	
C9	−0.1214 (3)	0.3095 (2)	0.62478 (7)	0.0547 (5)	
C10	−0.2118 (4)	0.1745 (2)	0.62336 (9)	0.0729 (7)	
C11	−0.0916 (4)	0.0699 (2)	0.64065 (10)	0.0856 (9)	
C12	0.1184 (4)	0.0977 (2)	0.65792 (9)	0.0724 (8)	
C13	0.2079 (3)	0.23213 (19)	0.66001 (7)	0.0562 (6)	
C14	0.0877 (3)	0.34244 (18)	0.64521 (6)	0.0477 (5)	
C15	0.3059 (2)	0.54985 (16)	0.69190 (6)	0.0451 (5)	
C16	0.3723 (2)	0.49081 (16)	0.74246 (6)	0.0429 (4)	
C17	0.6030 (2)	0.45802 (15)	0.74784 (5)	0.0371 (4)	
C18	0.8816 (2)	0.34649 (14)	0.79991 (5)	0.0341 (3)	
C19	0.9388 (2)	0.27621 (13)	0.84905 (5)	0.0340 (4)	
C20	0.6999 (2)	0.42049 (14)	0.89560 (5)	0.0340 (3)	
C21	0.6437 (2)	0.49041 (13)	0.84642 (5)	0.0337 (4)	
C22	0.9676 (2)	0.30256 (14)	0.94779 (6)	0.0382 (4)	
C23	1.1958 (3)	0.27466 (16)	0.95978 (6)	0.0461 (5)	
F3B	0.268 (2)	0.8830 (13)	0.4868 (3)	0.1469 (15)	0.127 (2)
F1B	0.304 (2)	0.9803 (8)	0.5651 (4)	0.1255 (9)	0.127 (2)
F2B	0.5195 (10)	0.8365 (13)	0.5404 (5)	0.0980 (7)	0.127 (2)
O1A	0.35437 (16)	0.18909 (10)	0.79446 (5)	0.0436 (3)	
O2A	0.60481 (16)	0.05328 (11)	0.77149 (6)	0.0524 (4)	
O3A	0.02774 (18)	−0.30289 (11)	0.78941 (6)	0.0588 (4)	
O4A	−0.25463 (17)	−0.18840 (12)	0.77479 (7)	0.0663 (5)	
C1A	0.4234 (2)	0.07131 (14)	0.78390 (6)	0.0364 (4)	
C2A	0.2834 (2)	−0.05762 (14)	0.78550 (6)	0.0385 (4)	
C3A	0.0781 (2)	−0.06083 (14)	0.78089 (6)	0.0383 (4)	
C4A	−0.0509 (2)	−0.19431 (14)	0.78216 (7)	0.0422 (4)	
O1B	0.24341 (15)	0.57369 (10)	0.90547 (4)	0.0412 (3)	
O2B	−0.01770 (15)	0.70858 (11)	0.92034 (5)	0.0456 (3)	
O3B	0.55830 (18)	1.06667 (11)	0.90607 (7)	0.0639 (5)	
O4B	0.84275 (17)	0.95113 (12)	0.91559 (7)	0.0623 (5)	
C1B	0.1684 (2)	0.69183 (13)	0.91202 (5)	0.0337 (4)	
C2B	0.3074 (2)	0.82060 (14)	0.91032 (6)	0.0391 (4)	
C3B	0.5119 (2)	0.82417 (13)	0.91468 (6)	0.0362 (4)	
C4B	0.6382 (2)	0.95740 (14)	0.91189 (7)	0.0424 (4)	
H1A	0.57380	0.31640	0.79770	0.0370*	
H5A	−0.07150	0.78460	0.49810	0.0790*	
H2A	1.00930	0.44800	0.89960	0.0370*	
H3A	0.38440	0.68180	0.60470	0.0620*	
H1	1.32630	0.45270	0.96450	0.0820*	
H12A	0.20020	0.02600	0.66820	0.0870*	
H13A	0.35030	0.24980	0.67140	0.0670*	
H15A	0.36010	0.64000	0.68930	0.0540*	
H16A	0.28670	0.40570	0.74390	0.0510*	
H6A	−0.29610	0.61380	0.52190	0.0780*	
H10A	−0.35280	0.15480	0.61080	0.0870*	
H11A	−0.15280	−0.01970	0.64060	0.1030*	
H18A	0.97870	0.42690	0.79980	0.0410*	
H18B	0.89440	0.28150	0.76880	0.0410*	
H19A	0.84760	0.19230	0.84800	0.0410*	
H19B	1.08220	0.24840	0.84970	0.0410*	
H20A	0.68780	0.48600	0.92660	0.0410*	
H20B	0.60140	0.34090	0.89590	0.0410*	
H21A	0.50090	0.51950	0.84580	0.0400*	
H21B	0.73650	0.57340	0.84710	0.0400*	
H22A	0.92550	0.36180	0.97760	0.0460*	
H22B	0.88450	0.21440	0.94430	0.0460*	
H23A	1.20680	0.20010	0.98240	0.0550*	
H23B	1.25010	0.24220	0.92690	0.0550*	
H16B	0.34780	0.55750	0.77210	0.0510*	
H17A	0.62940	0.39420	0.71750	0.0450*	
H17B	0.68950	0.54370	0.74820	0.0450*	
H2AA	0.34710	−0.14090	0.79010	0.0460*	
H3AA	0.01080	0.02140	0.77670	0.0460*	
H4A	−0.28400	−0.10740	0.77180	0.0990*	
H2BA	0.24320	0.90380	0.90580	0.0470*	
H3BA	0.58020	0.74270	0.91960	0.0430*	
H4B	0.87370	0.87110	0.91980	0.0930*	

### Atomic displacement parameters (Å^2^)

**Table:**

	*U*^11^	*U*^22^	*U*^33^	*U*^12^	*U*^13^	*U*^23^
S1	0.0438 (2)	0.0814 (3)	0.0728 (3)	−0.0019 (2)	0.0006 (2)	0.0191 (2)
F1A	0.170 (2)	0.1107 (15)	0.0830 (12)	−0.0800 (15)	−0.0226 (12)	0.0305 (11)
F2A	0.1009 (13)	0.0998 (12)	0.0986 (13)	−0.0100 (10)	0.0312 (10)	0.0223 (11)
F3A	0.1041 (14)	0.0976 (15)	0.267 (4)	0.0260 (11)	0.0317 (17)	0.126 (2)
O1	0.0495 (6)	0.0579 (7)	0.0531 (6)	−0.0124 (5)	−0.0078 (5)	0.0118 (5)
N1	0.0287 (5)	0.0269 (5)	0.0353 (5)	−0.0042 (4)	0.0045 (4)	0.0035 (4)
N2	0.0294 (5)	0.0255 (5)	0.0361 (5)	−0.0052 (4)	0.0029 (4)	0.0036 (4)
C1	0.0413 (8)	0.0470 (8)	0.0442 (7)	0.0051 (6)	0.0033 (6)	0.0112 (6)
C2	0.0482 (9)	0.0500 (8)	0.0446 (8)	0.0046 (7)	−0.0014 (6)	0.0097 (6)
C3	0.0498 (9)	0.0530 (9)	0.0525 (9)	0.0025 (7)	−0.0012 (7)	0.0155 (7)
C4	0.0674 (11)	0.0531 (10)	0.0563 (10)	0.0080 (8)	0.0036 (8)	0.0175 (8)
C5	0.0748 (13)	0.0643 (11)	0.0585 (10)	0.0112 (9)	−0.0086 (9)	0.0239 (9)
C6	0.0575 (11)	0.0723 (12)	0.0632 (11)	0.0093 (9)	−0.0139 (8)	0.0161 (9)
C7	0.0477 (9)	0.0575 (9)	0.0508 (9)	0.0049 (7)	−0.0001 (7)	0.0096 (7)
C8	0.0813 (14)	0.0591 (11)	0.0668 (12)	0.0025 (10)	0.0049 (10)	0.0258 (9)
C9	0.0535 (10)	0.0628 (10)	0.0470 (8)	−0.0071 (8)	0.0020 (7)	0.0115 (7)
C10	0.0738 (13)	0.0746 (13)	0.0660 (12)	−0.0245 (11)	−0.0044 (10)	0.0145 (10)
C11	0.112 (2)	0.0604 (12)	0.0783 (14)	−0.0293 (12)	−0.0090 (13)	0.0156 (11)
C12	0.0983 (17)	0.0516 (10)	0.0644 (12)	−0.0033 (10)	−0.0059 (11)	0.0127 (9)
C13	0.0656 (11)	0.0499 (9)	0.0516 (9)	0.0005 (8)	−0.0009 (8)	0.0088 (7)
C14	0.0534 (9)	0.0508 (9)	0.0386 (7)	−0.0028 (7)	0.0030 (6)	0.0084 (6)
C15	0.0456 (8)	0.0420 (8)	0.0477 (8)	0.0038 (6)	−0.0003 (6)	0.0109 (6)
C16	0.0417 (8)	0.0444 (8)	0.0425 (7)	0.0044 (6)	0.0009 (6)	0.0081 (6)
C17	0.0380 (7)	0.0363 (7)	0.0372 (7)	−0.0030 (5)	0.0030 (5)	0.0086 (5)
C18	0.0300 (6)	0.0335 (6)	0.0390 (6)	−0.0002 (5)	0.0084 (5)	0.0020 (5)
C19	0.0322 (7)	0.0283 (6)	0.0407 (7)	0.0011 (5)	0.0048 (5)	0.0003 (5)
C20	0.0301 (6)	0.0337 (6)	0.0382 (6)	−0.0014 (5)	0.0078 (5)	0.0021 (5)
C21	0.0320 (7)	0.0287 (6)	0.0396 (7)	0.0015 (5)	0.0045 (5)	0.0010 (5)
C22	0.0410 (8)	0.0324 (6)	0.0406 (7)	−0.0061 (5)	0.0016 (5)	0.0085 (5)
C23	0.0504 (9)	0.0390 (7)	0.0479 (8)	0.0036 (6)	−0.0044 (6)	0.0099 (6)
F3B	0.1041 (14)	0.0976 (15)	0.267 (4)	0.0260 (11)	0.0317 (17)	0.126 (2)
F1B	0.170 (2)	0.1107 (15)	0.0830 (12)	−0.0800 (15)	−0.0226 (12)	0.0305 (11)
F2B	0.1009 (13)	0.0998 (12)	0.0986 (13)	−0.0100 (10)	0.0312 (10)	0.0223 (11)
O1A	0.0335 (5)	0.0286 (5)	0.0689 (7)	−0.0024 (4)	0.0080 (4)	0.0069 (4)
O2A	0.0296 (5)	0.0373 (5)	0.0908 (9)	−0.0036 (4)	0.0150 (5)	0.0038 (5)
O3A	0.0432 (6)	0.0316 (5)	0.1042 (10)	−0.0014 (4)	0.0123 (6)	0.0179 (6)
O4A	0.0322 (6)	0.0350 (5)	0.1307 (13)	−0.0056 (4)	0.0127 (6)	0.0059 (7)
C1A	0.0283 (7)	0.0299 (6)	0.0501 (7)	−0.0038 (5)	0.0012 (5)	0.0071 (5)
C2A	0.0328 (7)	0.0274 (6)	0.0561 (8)	−0.0006 (5)	0.0046 (6)	0.0101 (5)
C3A	0.0327 (7)	0.0264 (6)	0.0558 (8)	−0.0015 (5)	0.0062 (6)	0.0055 (5)
C4A	0.0343 (7)	0.0295 (6)	0.0630 (9)	−0.0034 (5)	0.0104 (6)	0.0046 (6)
O1B	0.0353 (5)	0.0274 (4)	0.0616 (6)	−0.0028 (4)	0.0073 (4)	0.0085 (4)
O2B	0.0287 (5)	0.0359 (5)	0.0730 (7)	−0.0039 (4)	0.0117 (4)	0.0066 (5)
O3B	0.0389 (6)	0.0322 (5)	0.1227 (12)	−0.0019 (4)	0.0075 (6)	0.0216 (6)
O4B	0.0291 (6)	0.0342 (5)	0.1242 (12)	−0.0051 (4)	0.0110 (6)	0.0134 (6)
C1B	0.0293 (7)	0.0292 (6)	0.0423 (7)	−0.0045 (5)	0.0024 (5)	0.0076 (5)
C2B	0.0318 (7)	0.0266 (6)	0.0599 (8)	−0.0007 (5)	0.0047 (6)	0.0113 (6)
C3B	0.0301 (7)	0.0260 (6)	0.0522 (8)	−0.0024 (5)	0.0029 (5)	0.0071 (5)
C4B	0.0305 (7)	0.0296 (6)	0.0671 (9)	−0.0037 (5)	0.0051 (6)	0.0087 (6)

### Geometric parameters (Å, °)

**Table:**

S1—C7	1.757 (2)	C12—C13	1.382 (3)
S1—C9	1.758 (2)	C13—C14	1.403 (3)
F1A—C8	1.319 (3)	C15—C16	1.497 (2)
F1B—C8	1.301 (9)	C16—C17	1.5233 (18)
F2A—C8	1.325 (3)	C18—C19	1.5110 (18)
F2B—C8	1.289 (7)	C20—C21	1.5088 (18)
F3A—C8	1.297 (3)	C22—C23	1.506 (2)
F3B—C8	1.304 (8)	C3—H3A	0.9300
O1—C23	1.413 (2)	C5—H5A	0.9300
O1—H1	0.8200	C6—H6A	0.9300
O1A—C1A	1.2488 (17)	C10—H10A	0.9300
O2A—C1A	1.2548 (17)	C11—H11A	0.9300
O3A—C4A	1.2055 (18)	C12—H12A	0.9300
O4A—C4A	1.3053 (17)	C13—H13A	0.9300
O4A—H4A	0.8200	C15—H15A	0.9300
O1B—C1B	1.2567 (16)	C16—H16B	0.9700
O2B—C1B	1.2507 (16)	C16—H16A	0.9700
O3B—C4B	1.2083 (18)	C17—H17A	0.9700
O4B—C4B	1.3105 (17)	C17—H17B	0.9700
O4B—H4B	0.8200	C18—H18B	0.9700
N1—C21	1.4927 (16)	C18—H18A	0.9700
N1—C18	1.4944 (17)	C19—H19A	0.9700
N1—C17	1.5009 (17)	C19—H19B	0.9700
N2—C22	1.5074 (18)	C20—H20B	0.9700
N2—C20	1.4943 (17)	C20—H20A	0.9700
N2—C19	1.4965 (16)	C21—H21B	0.9700
N1—H1A	0.9100	C21—H21A	0.9700
N2—H2A	0.9100	C22—H22B	0.9700
C1—C14	1.482 (2)	C22—H22A	0.9700
C1—C15	1.340 (2)	C23—H23A	0.9700
C1—C2	1.483 (2)	C23—H23B	0.9700
C2—C7	1.401 (3)	C1A—C2A	1.5028 (19)
C2—C3	1.393 (3)	C2A—C3A	1.3084 (18)
C3—C4	1.387 (3)	C3A—C4A	1.4990 (19)
C4—C5	1.388 (3)	C2A—H2AA	0.9300
C4—C8	1.494 (3)	C3A—H3AA	0.9300
C5—C6	1.377 (3)	C1B—C2B	1.4984 (18)
C6—C7	1.395 (3)	C2B—C3B	1.3033 (18)
C9—C10	1.391 (3)	C3B—C4B	1.4956 (19)
C9—C14	1.394 (3)	C2B—H2BA	0.9300
C10—C11	1.379 (3)	C3B—H3BA	0.9300
C11—C12	1.375 (4)		
			
C7—S1—C9	100.77 (9)	C10—C11—H11A	120.00
C23—O1—H1	110.00	C11—C12—H12A	120.00
C4A—O4A—H4A	109.00	C13—C12—H12A	120.00
C4B—O4B—H4B	109.00	C14—C13—H13A	119.00
C17—N1—C21	112.51 (10)	C12—C13—H13A	119.00
C17—N1—C18	111.12 (10)	C1—C15—H15A	116.00
C18—N1—C21	108.81 (9)	C16—C15—H15A	116.00
C20—N2—C22	109.94 (10)	C15—C16—H16B	109.00
C19—N2—C22	112.35 (10)	C17—C16—H16B	109.00
C19—N2—C20	109.06 (10)	H16A—C16—H16B	108.00
C18—N1—H1A	108.00	C15—C16—H16A	109.00
C21—N1—H1A	108.00	C17—C16—H16A	109.00
C17—N1—H1A	108.00	N1—C17—H17B	109.00
C22—N2—H2A	108.00	N1—C17—H17A	109.00
C20—N2—H2A	108.00	H17A—C17—H17B	108.00
C19—N2—H2A	109.00	C16—C17—H17A	109.00
C14—C1—C15	123.86 (14)	C16—C17—H17B	109.00
C2—C1—C15	120.13 (15)	N1—C18—H18B	109.00
C2—C1—C14	115.96 (13)	C19—C18—H18B	109.00
C1—C2—C3	121.69 (16)	H18A—C18—H18B	108.00
C3—C2—C7	117.77 (16)	C19—C18—H18A	109.00
C1—C2—C7	120.50 (15)	N1—C18—H18A	109.00
C2—C3—C4	121.22 (17)	N2—C19—H19B	109.00
C3—C4—C5	120.28 (18)	C18—C19—H19B	109.00
C3—C4—C8	119.45 (17)	H19A—C19—H19B	108.00
C5—C4—C8	120.27 (18)	N2—C19—H19A	109.00
C4—C5—C6	119.44 (18)	C18—C19—H19A	109.00
C5—C6—C7	120.41 (18)	N2—C20—H20B	109.00
C2—C7—C6	120.82 (17)	C21—C20—H20A	109.00
S1—C7—C2	121.38 (14)	H20A—C20—H20B	108.00
S1—C7—C6	117.70 (15)	N2—C20—H20A	109.00
F1B—C8—F2B	108.3 (8)	C21—C20—H20B	109.00
F1B—C8—F3B	107.2 (7)	N1—C21—H21A	109.00
F2B—C8—F3B	108.1 (8)	C20—C21—H21B	109.00
F2A—C8—F3A	105.9 (2)	H21A—C21—H21B	108.00
F2A—C8—C4	112.33 (17)	C20—C21—H21A	109.00
F2B—C8—C4	115.3 (6)	N1—C21—H21B	109.00
F1B—C8—C4	109.6 (5)	N2—C22—H22B	109.00
F1A—C8—F2A	103.16 (19)	C23—C22—H22A	109.00
F1A—C8—F3A	109.0 (2)	H22A—C22—H22B	108.00
F1A—C8—C4	112.26 (18)	C23—C22—H22B	109.00
F3A—C8—C4	113.45 (18)	N2—C22—H22A	109.00
F3B—C8—C4	108.1 (6)	C22—C23—H23A	109.00
S1—C9—C14	121.65 (14)	O1—C23—H23A	109.00
S1—C9—C10	117.29 (16)	O1—C23—H23B	109.00
C10—C9—C14	121.06 (18)	H23A—C23—H23B	108.00
C9—C10—C11	119.8 (2)	C22—C23—H23B	109.00
C10—C11—C12	120.24 (19)	O2A—C1A—C2A	117.17 (12)
C11—C12—C13	120.02 (19)	O1A—C1A—O2A	123.74 (13)
C12—C13—C14	121.17 (19)	O1A—C1A—C2A	119.10 (12)
C1—C14—C9	120.51 (16)	C1A—C2A—C3A	124.55 (13)
C9—C14—C13	117.48 (17)	C2A—C3A—C4A	121.36 (12)
C1—C14—C13	122.01 (16)	O4A—C4A—C3A	116.89 (12)
C1—C15—C16	127.88 (14)	O3A—C4A—O4A	120.88 (13)
C15—C16—C17	112.50 (11)	O3A—C4A—C3A	122.23 (12)
N1—C17—C16	111.24 (11)	C1A—C2A—H2AA	118.00
N1—C18—C19	111.20 (10)	C3A—C2A—H2AA	118.00
N2—C19—C18	111.35 (10)	C4A—C3A—H3AA	119.00
N2—C20—C21	111.66 (10)	C2A—C3A—H3AA	119.00
N1—C21—C20	110.95 (10)	O1B—C1B—O2B	123.45 (12)
N2—C22—C23	114.20 (11)	O2B—C1B—C2B	117.50 (11)
O1—C23—C22	113.68 (13)	O1B—C1B—C2B	119.05 (12)
C4—C3—H3A	119.00	C1B—C2B—C3B	124.58 (12)
C2—C3—H3A	119.00	C2B—C3B—C4B	120.93 (12)
C6—C5—H5A	120.00	O3B—C4B—C3B	122.51 (12)
C4—C5—H5A	120.00	O4B—C4B—C3B	116.97 (12)
C7—C6—H6A	120.00	O3B—C4B—O4B	120.52 (13)
C5—C6—H6A	120.00	C1B—C2B—H2BA	118.00
C9—C10—H10A	120.00	C3B—C2B—H2BA	118.00
C11—C10—H10A	120.00	C2B—C3B—H3BA	120.00
C12—C11—H11A	120.00	C4B—C3B—H3BA	120.00
			
C9—S1—C7—C2	−30.94 (17)	C3—C4—C8—F1A	−29.5 (3)
C9—S1—C7—C6	152.85 (15)	C3—C4—C8—F2A	86.2 (2)
C7—S1—C9—C10	−152.63 (16)	C3—C4—C8—F3A	−153.7 (2)
C7—S1—C9—C14	28.28 (17)	C5—C4—C8—F1A	149.5 (2)
C18—N1—C17—C16	173.66 (11)	C5—C4—C8—F2A	−94.8 (2)
C21—N1—C17—C16	−64.06 (14)	C5—C4—C8—F3A	25.4 (3)
C17—N1—C18—C19	−177.84 (10)	C4—C5—C6—C7	1.7 (3)
C21—N1—C18—C19	57.75 (13)	C5—C6—C7—S1	173.90 (15)
C17—N1—C21—C20	178.72 (10)	C5—C6—C7—C2	−2.3 (3)
C18—N1—C21—C20	−57.70 (13)	S1—C9—C10—C11	178.60 (18)
C20—N2—C19—C18	55.99 (13)	C14—C9—C10—C11	−2.3 (3)
C22—N2—C19—C18	178.15 (10)	S1—C9—C14—C1	3.3 (2)
C19—N2—C20—C21	−56.27 (13)	S1—C9—C14—C13	−175.63 (13)
C22—N2—C20—C21	−179.87 (11)	C10—C9—C14—C1	−175.76 (17)
C19—N2—C22—C23	69.51 (14)	C10—C9—C14—C13	5.3 (3)
C20—N2—C22—C23	−168.83 (11)	C9—C10—C11—C12	−1.8 (4)
C14—C1—C2—C3	−146.11 (16)	C10—C11—C12—C13	2.6 (4)
C14—C1—C2—C7	36.3 (2)	C11—C12—C13—C14	0.6 (3)
C15—C1—C2—C3	36.4 (2)	C12—C13—C14—C1	176.62 (17)
C15—C1—C2—C7	−141.17 (16)	C12—C13—C14—C9	−4.5 (3)
C2—C1—C14—C9	−39.3 (2)	C1—C15—C16—C17	110.09 (16)
C2—C1—C14—C13	139.62 (16)	C15—C16—C17—N1	−177.47 (12)
C15—C1—C14—C9	138.10 (16)	N1—C18—C19—N2	−58.15 (14)
C15—C1—C14—C13	−43.0 (2)	N2—C20—C21—N1	58.42 (13)
C2—C1—C15—C16	173.11 (14)	N2—C22—C23—O1	81.02 (15)
C14—C1—C15—C16	−4.1 (2)	O1A—C1A—C2A—C3A	22.3 (2)
C1—C2—C3—C4	−175.68 (17)	O2A—C1A—C2A—C3A	−157.25 (16)
C7—C2—C3—C4	2.0 (3)	C1A—C2A—C3A—C4A	179.16 (14)
C1—C2—C7—S1	2.1 (2)	C2A—C3A—C4A—O3A	2.8 (3)
C1—C2—C7—C6	178.20 (16)	C2A—C3A—C4A—O4A	−176.90 (16)
C3—C2—C7—S1	−175.58 (14)	O1B—C1B—C2B—C3B	−16.6 (2)
C3—C2—C7—C6	0.5 (3)	O2B—C1B—C2B—C3B	162.92 (15)
C2—C3—C4—C5	−2.7 (3)	C1B—C2B—C3B—C4B	179.28 (14)
C2—C3—C4—C8	176.38 (17)	C2B—C3B—C4B—O3B	0.7 (3)
C3—C4—C5—C6	0.8 (3)	C2B—C3B—C4B—O4B	−178.69 (16)
C8—C4—C5—C6	−178.23 (18)		

### Hydrogen-bond geometry (Å, °)

**Table:**

*D*—H···*A*	*D*—H	H···*A*	*D*···*A*	*D*—H···*A*
O1—H1···O1B^i^	0.82	2.05	2.8365 (16)	162
N1—H1A···O1A	0.91	1.81	2.7055 (15)	168
N1—H1A···O2A	0.91	2.57	3.2580 (15)	133
N2—H2A···O1B^i^	0.91	1.86	2.7572 (15)	167
N2—H2A···O2B^i^	0.91	2.52	3.2230 (15)	134
O4A—H4A···O2A^ii^	0.82	1.73	2.5406 (16)	167
O4B—H4B···O2B^i^	0.82	1.74	2.5497 (16)	168
C2A—H2AA···O3A	0.93	2.51	2.8251 (17)	100
C2B—H2BA···O3B	0.93	2.50	2.8165 (17)	100
C16—H16B···O3A^iii^	0.97	2.56	3.2782 (19)	131
C17—H17B···O4A^iv^	0.97	2.59	3.4520 (19)	148
C19—H19A···O2A	0.97	2.57	3.2871 (18)	131
C19—H19B···O1A^i^	0.97	2.41	3.2461 (17)	144
C20—H20A···O1^v^	0.97	2.44	3.3877 (18)	167
C21—H21A···O1B	0.97	2.41	3.2098 (16)	140
C21—H21B···O2B^i^	0.97	2.51	3.2367 (17)	132
C22—H22B···O3B^vi^	0.97	2.51	3.3848 (18)	150
C22—H22B···O4B^vi^	0.97	2.55	3.4236 (18)	150

Symmetry codes: (i) *x*+1, *y*, *z*; (ii) *x*−1, *y*, *z*; (iii) *x*, *y*+1, *z*; (iv) *x*+1, *y*+1, *z*; (v) −*x*+2, −*y*+1, −*z*+2; (vi) *x*, *y*−1, *z*.
